# Downregulation of ADAMTS3 Suppresses Stemness and Tumorigenicity in Glioma Stem Cell

**DOI:** 10.1111/cns.14052

**Published:** 2022-12-13

**Authors:** Hyun‐Jin Kim, Hang Yeon Jeong, Don Carlo Batara, Changjong Moon, Seongsoo Lee, Suk Jun Lee, Sang‐Ik Park, Moon‐Chang Choi, Sung‐Hak Kim

**Affiliations:** ^1^ Department of Animal Science, College of Agriculture and Life Sciences Chonnam National University Gwangju Korea; ^2^ Department of Veterinary Anatomy and Animal Behavior College of Veterinary Medicine and BK21 FOUR Program, Chonnam National University Gwangju Korea; ^3^ Gwangju Center Korea Basic Science Institute Gwangju Korea; ^4^ Department of Biomedical Laboratory Science College of Health & Medical Sciences, Cheongju University Chungbuk Korea; ^5^ Laboratory of Veterinary Pathology College of Veterinary Medicine and BK21 Plus Project Team, Chonnam National University Gwangju Korea; ^6^ Department of Biomedical Science Chosun University Gwangju Korea

**Keywords:** ADAMTS3, GBM, glioma stem cell, tumor formation

## Abstract

**Aims:**

Glioblastoma multiforme (GBM) is the most aggressive type of human brain tumor, with a poor prognosis and a median overall survival of fewer than 15 months. Glioma stem cells (GSCs) have recently been identified as a key player in tumor initiation and therapeutic resistance in GBM. ADAMTS family of metalloproteinases is known to cleave a wide range of extracellular matrix substrates and has been linked to tissue remodeling events in tumor development. Here, we investigate that ADAMTS3 regulates GSC proliferation and self‐renewal activities, and tumorigenesis in orthotopic xenograft models.

**Methods:**

*ADAMTS3* mRNA expression levels in normal human astrocyte (NHA), glioma, and GSCs cell lines were compared. After knockdown of *ADAMTS3*, alamarBlue assay, in vitro limiting dilution, and orthotopic xenograft assays were performed. To investigate the tumor‐associated roles of ADAMTS3, several statistical assays were conducted using publicly available datasets.

**Results:**

*ADAMTS3* level was remarkably higher in GSCs than in NHA, glioma cell lines, and their matched differentiated tumor cells. Interestingly, knockdown of *ADAMTS3* disrupted GSC's proliferation, self‐renewal activity, and tumor formation in vivo. Furthermore, ADAMTS3 could be used as an independent predictor of malignancy progression in GBM.

**Conclusion:**

We identified ADAMTS3 as a potential therapeutic target for GBM.

## Graphical abstract

ADAMTS3 mRNA level is significantly higher in GSCs.

Knockdown of ADAMTS3 suppresses GSC's proliferation, self‐renewal activity, and tumor formation in vivo.

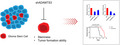

## 1 INTRODUCTION

Glioblastoma (GBM) is one of the most deadly and difficult‐to‐treat brain tumors in the human central nervous system (CNS). Despite aggressive treatments such as surgical resection, radiation, and chemotherapy, GBM patients have a median overall survival of 15 months after diagnosis.^1^, ^2^ It was recently found that it preserves a cell population known as glioma stem cells (GSCs), which are capable of tumor initiation, treatment resistance, and invasion, as well as the ability to develop intratumoral heterogeneity.^3^, ^4^ As such, identifying the appropriate therapeutic target for preventing GSCs has become essential.

A Disintegrin and Metalloproteinase with Thrombospondin Motifs (ADAMTS) enzymes are secreted matrix‐associated zinc metalloendopeptidases that play a range of roles in tissue morphogenesis, pathological remodeling, inflammation, and vascular biology. The human family consists of 19 members, which can be divided into sub‐groups based on their known substrates. Control of the extracellular matrix (ECM) function and structure is a central aspect of the ADAMTS domain, as evidenced by the actions of procollagen‐N‐propeptidases in collagen fibril assembly and aggrecanases in the modification or cleavage of ECM proteoglycans.^5^, ^6^ Inherited genetic diseases result from defects in certain family members, whereas abnormal expression or function in others is linked to arthritis, cancer, and cardiovascular disease.^7^ Multiple ADAMTSs from various sub‐groups have been shown to have beneficial or detrimental effects on tumor initiation and metastasis, with both metalloproteinase‐dependent and ‐independent effects.^8^

In comparison to other ADAMTS families, the role of ADAMTS3 is not fully known. ADAMTS3 is a procollagen N‐propeptidase that is essential for the maturation of triple‐helical collagen fibrils. It is expressed particularly in cartilage during embryogenesis and adulthood, and it efficiently processes the procollagen II aminopropeptide in vitro.^5^, ^9^ According to an in situ hybridization (ISH) study, ADAMTS3 was previously reported to be extensively expressed in the developing mouse brain's hindbrain region, as well as connective tissues such as bone and tendon.^10^, ^11^ In osteosarcoma models, ADAMTS3 could modulate collagen maturation and biosynthesis by a transcription factor SP1.^12^ However, the function and significance of ADAMTS3 in GBM remain unknown, thus, more research is required.

To investigate the link between ADAMTS3 expression and GBM malignancy, we compared the *ADAMTS3* expression levels using Gene Expression Omnibus (GEO) (GSE4536) dataset and employed univariate and multivariate analysis using the Gravendeel dataset. Also, we examined the effect of *ADAMTS3* knockdown on self‐renewal and tumor formation ability in vitro and in vivo of GSCs isolated from primary GBM tissues.

## 2 MATERIALS AND METHODS

### 2.1 Cell culture and reagents

Normal human astrocytes (NHA) (ScienCell Research Laboratories, CA, USA) were maintained in an astrocyte medium containing 10% fetal bovine serum (FBS; HyClone) and 1% penicillin–streptomycin solution (100X, Welgene). Glioma cells (A172, A1207, U87MG, and LN229) were maintained in Serum media composing Dulbecco's modified medium (DMEM/F12; Welgene) containing 10% Fetal Bovine Serum (FBS; HyClone) and 1% penicillin–streptomycin solution (100X, Welgene). A Neurobasal Media (NBE) composed of DMEM/F12 medium (Welgene) supplemented with 2% B27 (Gibco), 1% penicillin–streptomycin solution (100X, Welgene), epidermal growth factor (20 ng/ml; R&D Systems), and basic fibroblast growth factor (20 ng/ml; R&D Systems), were used to maintain glioma stem cells (GSC11, GSC20, GSC23, and GSC267) received from the University of Texas MD Anderson Cancer Center.^13^

### 2.2 Quantitative reverse transcription‐PCR (RT‐qPCR)

Total RNA was extracted using the RiboEx reagent (GeneAll) and purified using the HybridR kit (GeneAll), as directed by the manufacturer. Thermo Scientific RevertAid FirstStrand cDNA Synthesis Kit (Thermo Fisher Scientific) was used to convert 500 ng of total RNA to complementary DNA (cDNA). The quantitative Real‐Time PCR (qPCR) was performed using TB Green Premix Ex Taq (Takara Bio) on a CFX96 real‐time polymerase chain reaction detection system (BioRad Laboratories). The 2^−ΔΔCt^ method was used to calculate the cycle threshold (Ct) values from the qPCR results. Primer sequences (5′ to 3′) used for qPCR were as follows: *18 S* (loading control): forward (F), CAGCCACCCGAGATTGAGCA and reverse (R), TAGTAGCGACGGGCGGTGTG; *ADAMTS3*: F, CAGCAGCGAAACTCCCACTTTG and R, GCGTTCCATCATGCACCAGTTG; *CD15*: F, TTGGGACCTCCTAGTTCCAC and R, TGTAAGGAAGCCACATTGGA; *CD133*: F, CAGGTAAGAACCCGGATCAA and R, TCAGATCTGTGAACGCCTTG; *GFAP*: F, GGAACATCGTGGTGAAGACC and R, AGAGGCGGAGCAACTATCCT; *SOX2*: F, GCTACAGCATGATGCAGGACCA and R, TCTGCGAGCTGGTCATGGAGTT; *NES*: F, TCAAGATGTCCCTCAGCCTGGA and R, AAGCTGAGGGAAGTCTTGGAGC; *OLIG2*: F, ATGCACGACCTCAACATCGCCA and R, ACCAGTCGCTTCATCTCCTCCA.

### 2.3 Dataset analysis using public datasets

Gene Expression Omnibus (GSE4536) datasets were used to compare the *ADAMTS3* mRNA expression in different glioma stem cell cultures. The gene expressions were normalized using dChip invariant method and PM‐MM difference model was used to obtain the expression values. Also, the Gravendeel dataset obtained from the Gliovis website (http://gliovis.bioinfo.cnio.es/) was used to analyze ADAMTS3 gene expression profiles and correlation analysis with patient survival and other GSC‐related genes.

### 2.4 Lentiviral infection for ADAMTS3 knockdown

Lentiviral vectors expressing nontarget shRNA (shNT), and shRNA construct targeting *ADAMTS3* (TRCN0000050569, TRCN0000050572; Sigma‐Aldrich) were used to silence *ADAMTS3* expression. For packaging the lentivirus, 293FT (Invitrogen) cells were transfected using the CalPhos Mammalian Transfection Kit (Takara Bio), the lentivirus was harvested at 72 h after transfection and concentrated 100‐fold using Lenti‐X concentrator (Takara Bio). Lentivirus infection was performed according to the manufacturer's protocol.

### 2.5 Gene ontology (GO) and Kyoto encyclopedia of genes and genomes (KEGG) pathway analysis

GO and KEGG pathway analyses were performed using the ShinyGO (version 0.75. http://bioinformatics.sdstate.edu/go/) to determine the genes that are coupregulated with *ADAMTS3* (log2 FC ≥1.0 and *p* ≤ 0.001) in Gravendeel dataset.^14^

### 2.6 Cell viability

Cell viability of GSCs after shRNA transduction was assessed using the alamarBlue® cell viability assay (Invitrogen, USA). shRNA‐transduced GSCs were seeded onto 96‐well plates at a density of 3000 cells/well (*n* = 6). After 72 h of incubation, 10 μl of alamarBlue reagent was added to each well and incubated for another 6 h. At the end of the incubation, Fluorescence was measured at a wavelength of 590 nm in a Synergy HTX Multi‐Mode Reader (BioTek Instruments Inc).

### 2.7 In vitro limiting dilution assay

Using an in vitro limiting dilution assay, shRNA‐transduced GSCs were seeded in a 96‐well plate in a decreasing cell number (50, 25, 12, 6, 3, and 1 cell/well) to determine their tumorsphere formation ability. The frequency of tumorsphere formation ability of GSCs was determined using Extreme Limiting Dilution Analysis (ELDA) software available at http://bioinf.wehi.edu.au/software/elda.^15^

### 2.8 In vivo orthotopic implantation

For in vivo orthotopic implantation, shNT or shADAMTS3 treated GSCs (5 × 10^5^ cells) were intracranially injected into the nude mice brains at coordinates: 2 mm right and 1.0 mm anterior of the bregma using a stereotaxic instrument (*n* = 6 mice per group). Mice that had lost more than 30% of their body weight were sacrificed. The Kaplan–Meier method was used to present overall survival curves. For tissue histology analysis, one mouse was simultaneously sacrificed in the control and the experimental groups after 1 month of GSC injection. All mice experiments were authorized by Chonnam National University's Animal Care Committee following the Republic of Korea government and institutional standards and laws (CNU IACUC‐YB‐2021‐99).

### 2.9 In vivo bioluminescence imaging

For imaging procedures, isoflurane at a concentration of 2% was used to maintain the anesthesia of nude mice. Following intraperitoneal injection of 150 mg/kg D‐luciferin, mice were imaged with 10 min of exposure. Total flux values were determined by luminescent regions of interest (ROI) of each mouse and are presented in radiance (p/sec/cm^2^/sr).

### 2.10 Statistical analysis

For statistical analyses, Microsoft Excel, SPSS Ver.20, and GraphPad Prism Ver.8.0 were used. Statistical significance between and among groups were assessed by a two‐tailed *t*‐test and one‐way ANOVA, respectively, followed by Tukey's multiple comparison test. In the univariate and multivariate Cox regression analyses, the *p‐Value* <0.05 was indicated as statistically significant. The heat maps were created using GraphPad Prism Ver.8.0. Also, for correlation analysis, the Pearson Correlation Coefficient (*R‐value*) between *ADAMTS3* and each GSC subtype marker was calculated using GraphPad Prism Ver.8 software.

## 3 RESULTS

### 3.1 ADAMTS3 mRNA is elevated under GSCs culture condition

**FIGURE 1:**
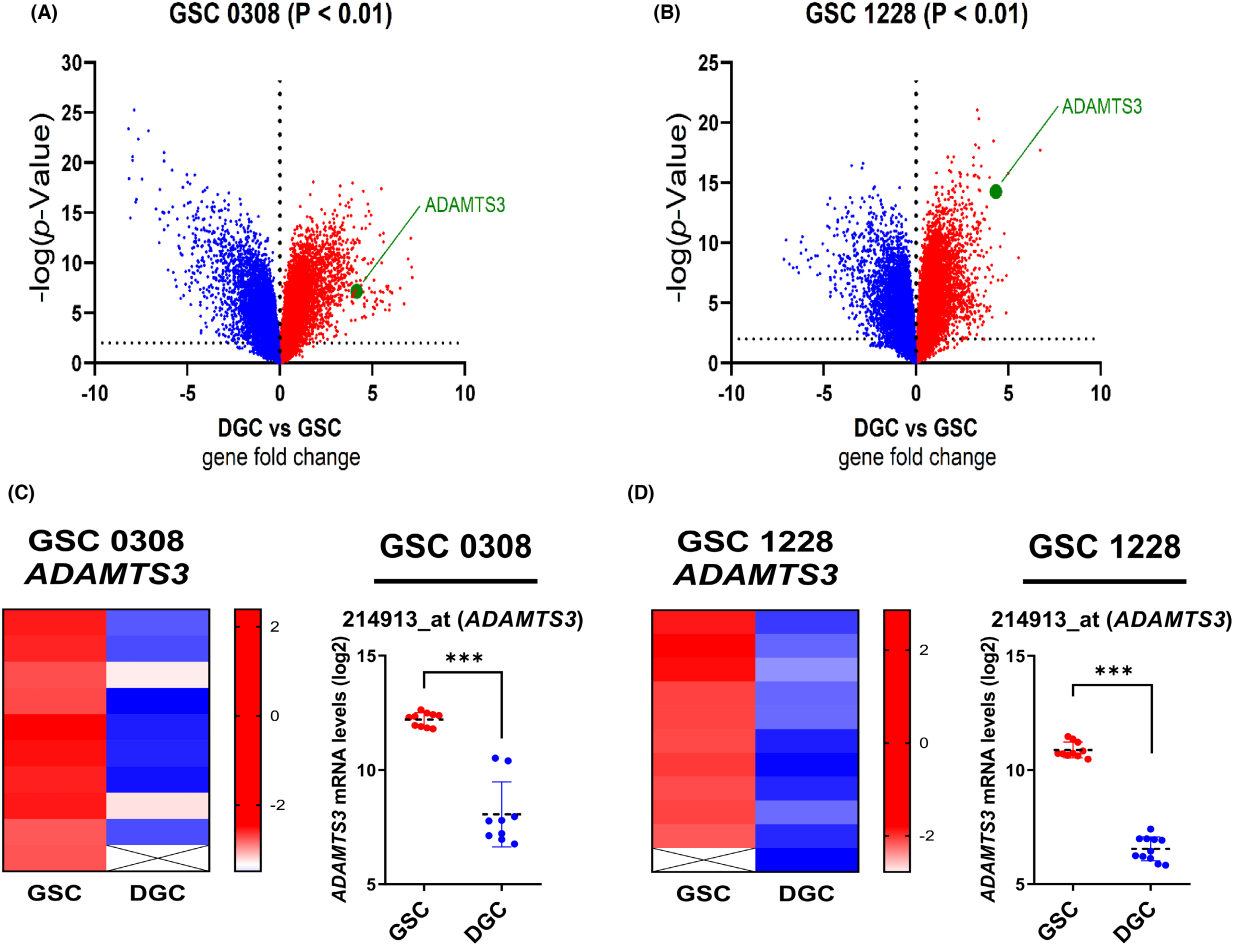
*ADAMTS3* mRNA is elevated under GSCs culture condition. (A) and (B) Volcano plot describes the different expression level in GSCs cultured under NBE and Serum conditions according to GSE4536 dataset. (A), 0308 cell; (B), 1228 cell. (C) and (D) The comparison of *ADAMTS3* expression in GSCs cultured under NBE and FBS conditions according to multiple probe sets of GSE4536 dataset. (C), 0308 cell; (D), 1228 cell. Data are means ± SEM (NBE, *n* = 10 or 11; FBS, *n* = 11 or 10). ***p < 0.001.

According to a previous report, when compared to serum‐cultured differentiated GSCs, NBE (Neurobasal media with EGF and bFGF) cultured undifferentiated GSCs had more robust tumorigenic potential, heterogeneous morphology, and indefinite self‐renewal ability.^16^ Using data from a publicly available dataset (GSE4536), we first investigated the expression levels of the ADAMTS3 gene in GSCs maintained in NBE (serum‐free DMEM/F12 media supplemented with basic bFGF and EGF) media and Serum media (Figure 1A,B). The levels of *ADAMTS3* expression reduced significantly when cells were differentiated in a serum‐containing medium (Figure 1C,D). These findings suggest that *ADAMTS3* is associated with GSC's stemness.

### 3.2 ADAMTS3 is highly expressed in glioma stem cells

**FIGURE 2:**
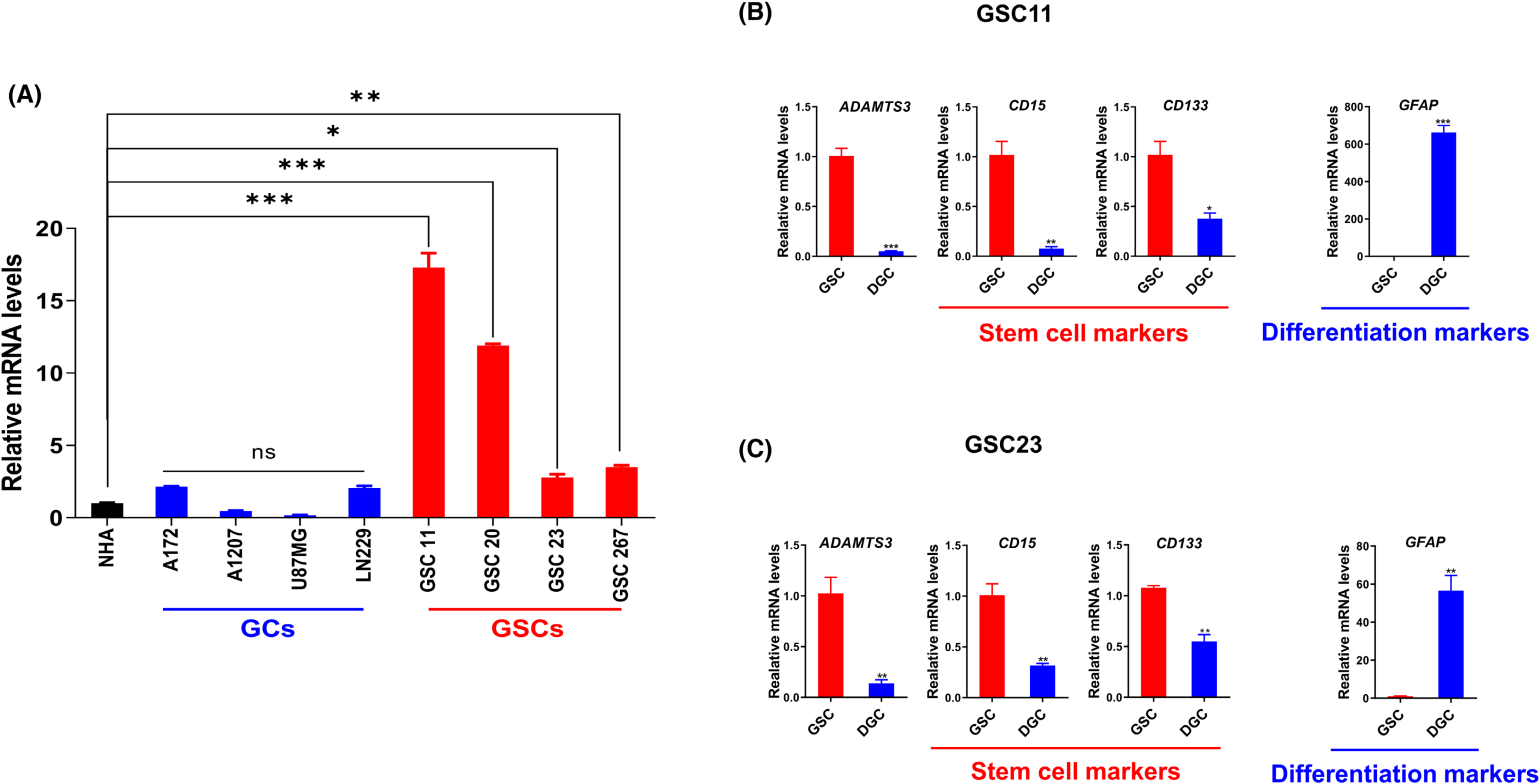
*ADAMTS3* is highly expressed in glioma stem cells. (A) RT‐qPCR analysis of mRNA expression of *ADAMTS3* in normal human astrocyte (NHA), glioma cells, and cancer stem cells. Data are means ± SEM (*n* = 3). *p < 0.05, **p < 0.01, ***p < 0.001, ns (nonsignificant). (B) and (C) RT‐qPCR analysis of *ADAMTS3*, *CD15*, *CD133*, and *GFAP* mRNA expressions in NBE‐ and Serum‐cultured GSCs. Data are means ± SEM (*n* = 3). *p < 0.05, **p < 0.01, ***p < 0.001. (B), GSC11; (C), GSC23.

To validate *ADAMTS3* expression levels in our cell lines, *ADAMTS3* mRNA expression levels in NHA, glioma cell lines (A172, A1207, U87MG, and LN229), and patient‐derived GSCs (GSC11, 20, 23, and 267) were examined. *ADAMTS3* was highly upregulated in GSCs compared to NHA and glioma cell lines, according to qRT‐PCR analysis (Figure 2A). We also confirmed that there is a substantial increase of *ADAMTS3* mRNA expression in GSCs NBE cultures relative to matched differentiated GSCs in serum‐containing media. *CD133*, *CD15*, and *GFAP* were used to validate the cellular phenotype (Figure 2B,C). Hence, these results show that *ADAMTS3* is significantly expressed in GSCs.

### 3.3 Silencing of ADAMTS3 suppresses the stemness of GSC

**FIGURE 3:**
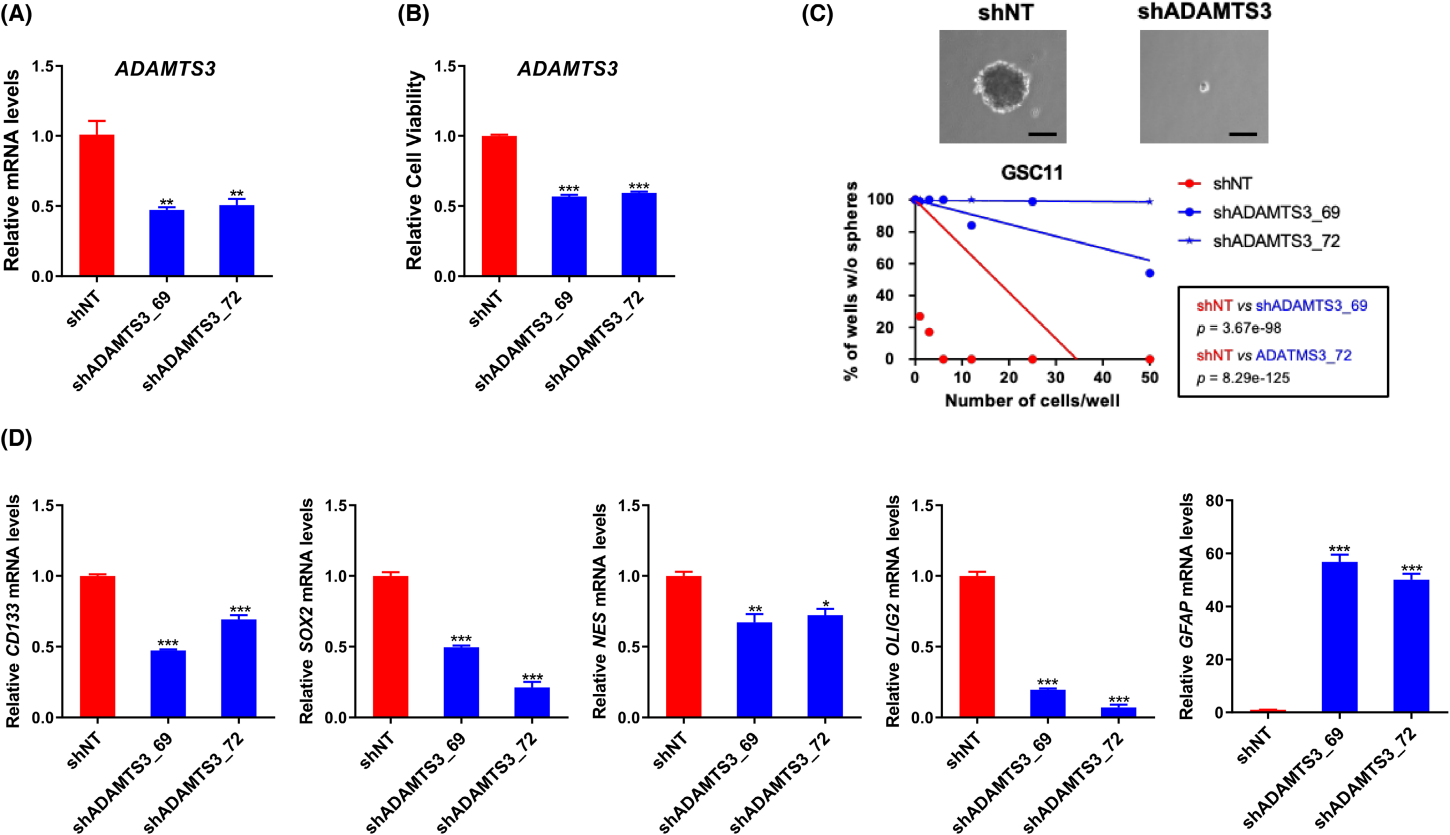
Silencing of *ADAMTS3* suppresses the stemness of GSC. (A) RT‐qPCR analysis showing *ADAMTS3* knockdown after transduction with shRNA in GSC11. Data are means ± SEM (*n* = 3). ***p < 0.001. (B) Cell viability in sh*ADAMTS3* treated‐GSC11. Data are means ± SEM (*n* = 3). *p < 0.05, **p < 0.01. (C) Effects of *ADAMTS3* knockdown on neurosphere formation and self‐renewal ability. Scale bars represent 100 μm. (D) Glioma stem cell marker and differentiation marker expression after *ADAMTS3* knockdown in GSC11. Data are means ± SEM (*n* = 3). *p < 0.05, **p < 0.01, ***p < 0.001.

To understand the function of ADAMTS3 in GSCs, we employed an shRNA lentivirus to knockdown *ADAMTS3* in GSC11 cell line. shRNA knockdown significantly reduced *ADAMTS3* mRNA levels by 50% in GSC11 cell line, based on qRT‐PCR analysis (Figure 3A). The effects of *ADAMTS3* knockdown on cell viability and tumorsphere forming capacity were then investigated. *ADAMTS3* knockdown significantly reduced cell viability and tumorsphere development in GSC11 compared to nontargeting shRNA (Figure 3B,C).^17^ Furthermore, after *ADAMTS3* knockdown, the expression levels of GSC‐related stemness were significantly reduced. Surprisingly, one of the differentiation markers, GFAP, showed increased expression following ADAMTS3 knockdown. (Figure 3D). These findings suggest that ADAMTS3 is involved in the maintenance of GSC stemness.

### 3.4 Silencing of ADAMTS3 inhibits tumorigenesis in GSC

**FIGURE 4:**
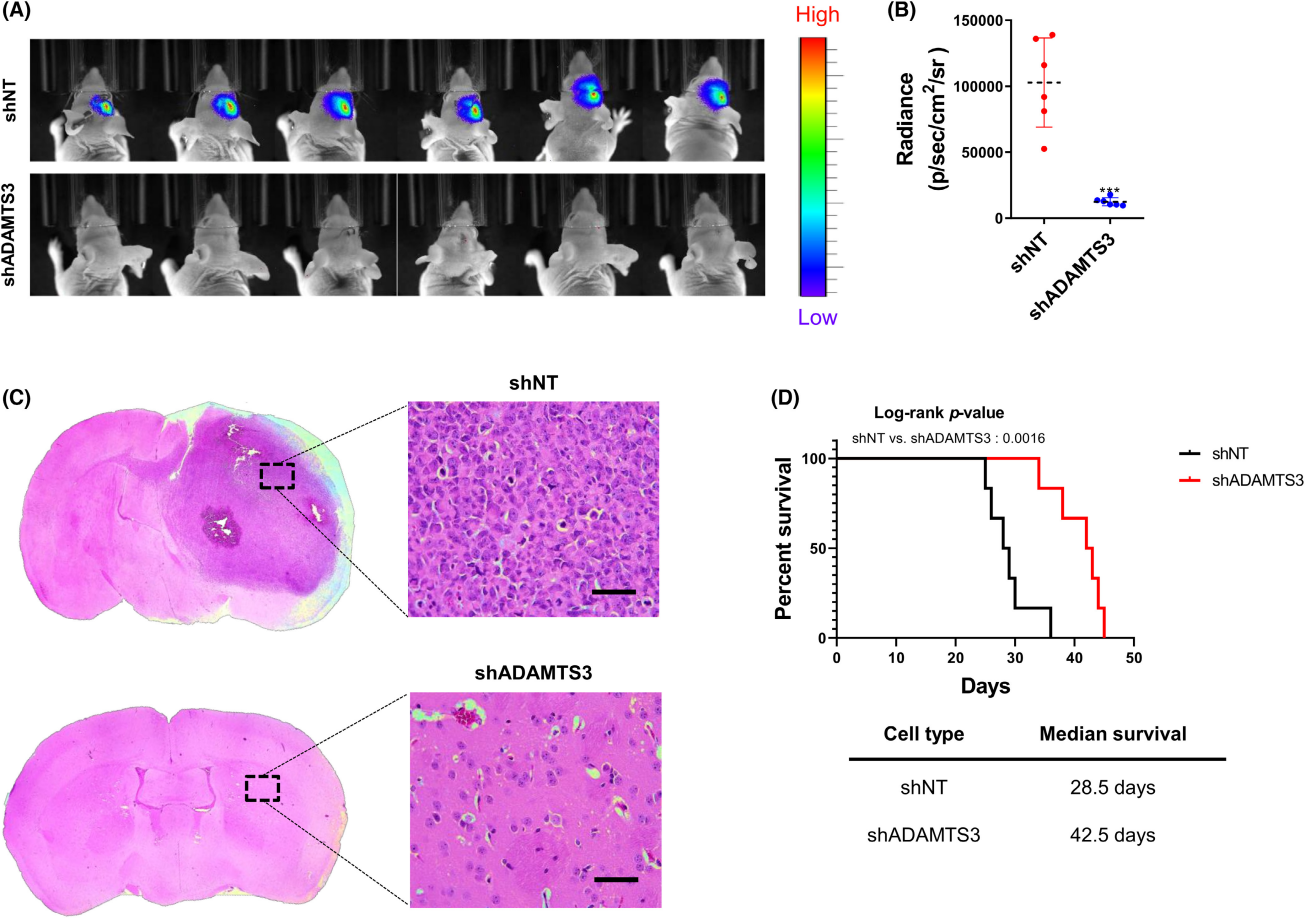
Silencing of *ADAMTS3* inhibits tumorigenesis in GSC. (A) and (B) In vivo bioluminescent imaging was performed on nude mice bearing intracranial xenografts derived from shADAMTS3 treated GSC11. Data are means ± SEM (*n* = 6). ***p < 0.001. (C) Representative images of H&E‐stained coronal sections of tumor‐bearing brains harvested after implantation of shNT(Nontarget) and shADAMTS3 treated GSC11. Scale bars represent 100 μm. (D) Kaplan–Meier survival curves of nude mice bearing intracranial tumors derived from shNT and shADAMTS3 treated GSC11.

Intracranial mouse models were used to investigate the effect of suppressing *ADAMTS3* expression in GSC11 spheres in vivo. GSC from patient‐derived patients were transduced with a constitutive luciferase reporter to enable continuous monitoring of tumor volume (Figure 4A,B). When compared to shNT‐control infected GSC11 spheres, mice infected with *ADAMTS3* knockdown had significantly reduced tumor formation and had significantly longer median survival rates (Figure 4C,D). Hence, these data indicate that ADAMTS3 is required for in vivo growth of GSC.

### 3.5 ADAMTS3 is preferentially expressed in Grade IV tumors and correlated with poor prognosis

**FIGURE 5:**
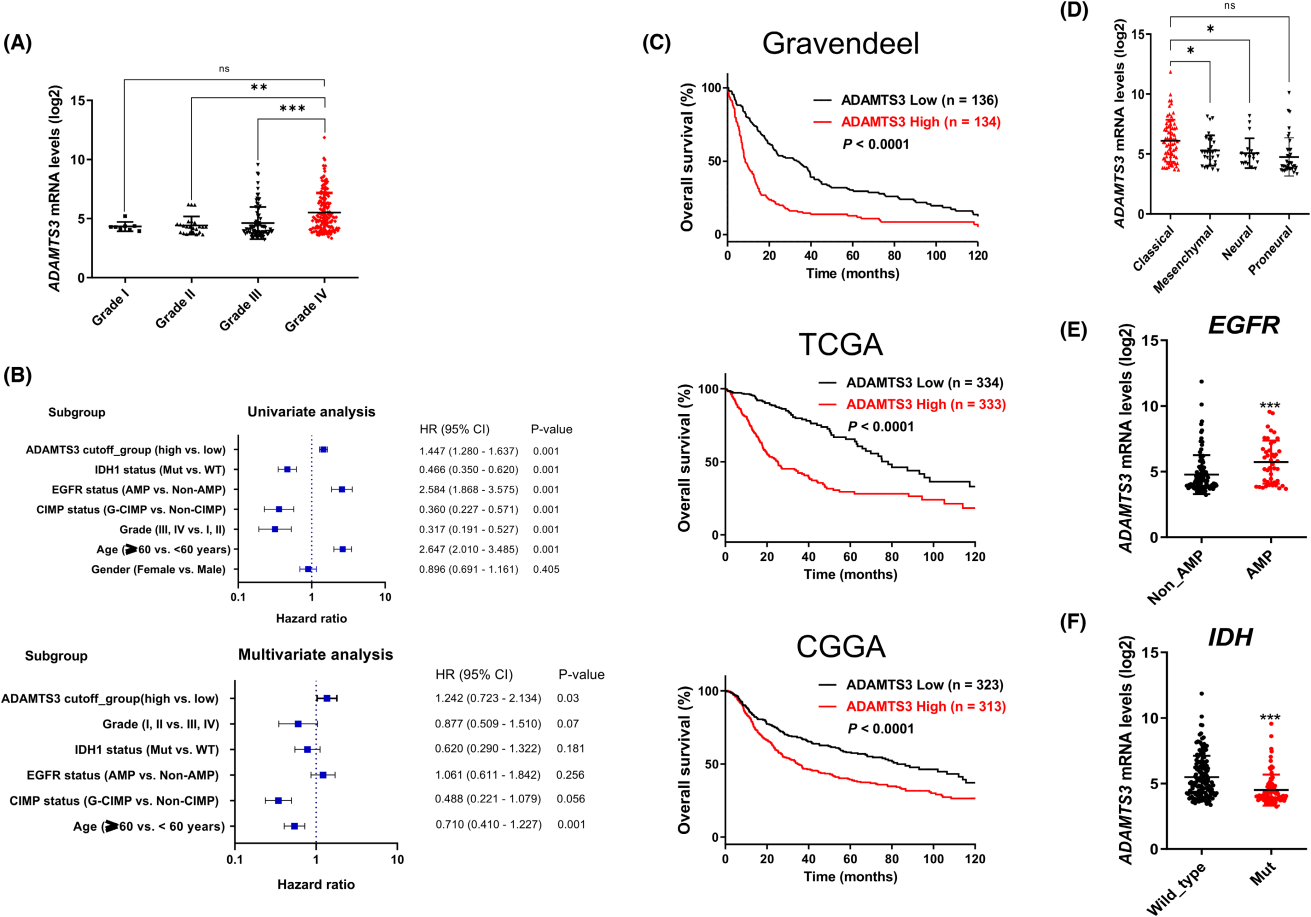
*ADAMTS3* is preferentially expressed in Grade IV tumors and correlated with poor prognosis. (A) Comparison of *ADAMTS3* mRNA levels by glioma grade using Gravendeel dataset. Data are means ± SEM (Grade I, *n* = 8; Grade II, *n* = 24; Grade III, *n* = 85; Grade IV, *n* = 159). **p < 0.01, ***p < 0.001. (B) Univariate and multivariate Cox regression of *ADAMTS3* expression for overall survival in glioma patients. (C) Kaplan–Meier survival curves of 270 all subtype patients (*ADAMTS3* high, *n* = 135; *ADAMTS3* low, *n* = 136, *p* < 0.0001) in the Gravendeel dataset. Kaplan–Meier survival curves of 667 all subtype patients (*ADAMTS3* high, *n* = 333; *ADAMTS3* low, *n* = 334, *p* < 0.0001) in The Cancer Genome Atlas (TCGA) dataset. Kaplan–Meier survival curves of 636 all subtype patients (*ADAMTS3* high, *n* = 313; *ADAMTS3* low, *n* = 323, *p* < 0.0001) in Chinese Glioma Genome Atlas (CCGA) dataset. (D) Comparing ADAMTS3 mRNA levels in Glioblastoma multiforme subtypes from the Gravendeel subtype_Verhaak_2010 dataset (Classical, *n* = 72; Mesenchymal, *n* = 30; Neural, *n* = 19; Proneural, *n* = 38). *p < 0.05, ns (nonsignificant). (E) Comparison of ADAMTS3 levels between EGFR non‐AMP and EGFR AMP groups. ***p < 0.001. (F) Comparison of ADAMTS3 levels between IDH wild type and IDH mutation groups. ***p < 0.001.

Further, we examined the correlation between *ADAMTS3* expression and glioma patient prognosis using Gravendeel datasets. Accordingly, the *ADAMTS3* level was more highly expressed in Grade IV (GBM) samples than in low‐grade glioma samples (Figure 5A). Univariate analysis showed significant correlations of overall patient survival with *ADAMTS3* expression level, *IDH1* status, *EGFR* status, *CIMP* status, grade, and age (all *p‐Value* = 0.001) (Figure 5B). Moreover, High *ADAMTS3* expression (*p* = 0.03) and age (*p* = 0.001) were found to be factors influencing glioma patients' survival in multivariate analysis. (Figure 5B). Based on the Kaplan–Meier survival curve, patients with higher *ADAMTS3* expression had shorter overall survival. In addition to the Gravendeel dataset, the inverse correlation between *ADAMTS3* expression and overall survival was also present in The Cancer Genome Atlas (TCGA) and Chinese Glioma Genome Atlas (CGGA) datasets (Figure 5C). We employed three previously described GBM subtypes to investigate the preferential expression of *ADAMTS3*. According to the Gravendeel dataset, the classical subtype has much higher *ADAMTS3* expression than the mesenchymal and neural subtypes. (Figure 5D). In addition, the difference between *EGFR* nonamplification and *EGFR* amplification was significant, with EGFR vIII amplification being considered a genetic change in the classical subtype. (Figure 5E). Moreover, we confirmed ADAMTS3 expression between wild type and IDH mutant. As we expected, ADAMTS3 expression was lower in mutant than wild type, which was consistent with our other results. As such, these data demonstrate that *ADAMTS3* is correlated with poor prognosis in glioma patients.

### 3.6 Identification of coexpressed Genes with ADAMTS3 in GBM Samples

**FIGURE 6:**
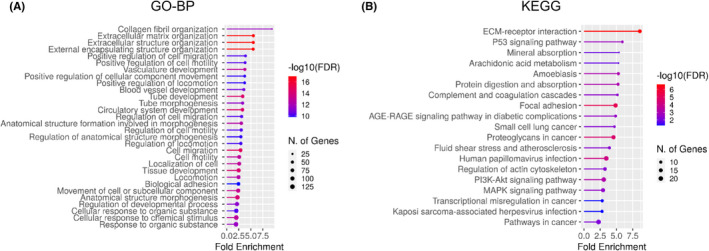
Identification of coexpressed Genes with *ADAMTS3* in GBM Samples. (A) and (B) Lollipop plots present enrichment pathways of genes coexpressed with *ADAMTS3* in Gravendeel dataset. The *p*‐Value of all coexpressed genes is less than 0.05. Among the total number of genes annotated in the network, the ‘fold enrichment’ of genes is assigned to a category.

Finally, we categorized the down‐ and upregulated genes that were coexpressed with *ADAMTS3* to better understand ADAMTS3's signaling pathways and biological processes. Using the Gravendeel dataset, we found 444 coexpressed genes that were upregulated with *ADAMTS3* (logFC >1, *p* < 0.001). Using ShinyGO v0.75, these positively correlated genes were analyzed for GO biological processes and KEGG pathways (Figure 6A,B). Coexpressed genes were involved in an extracellular matrix organization, vasculature development, tissue development, and blood vessel development in terms of gene ontology biological processes. A previous study has reported that the ADAMTS3 signaling pathway is involved in lymphangiogenesis and angiogenesis, implying a possible role of ADAMTS3 in GBM vascular development.^10^, ^18^, ^19^ Furthermore, the protein digestion and absorption, ECM‐receptor interaction, PI3K‐Akt signaling pathway, focal adhesion, MAPK signaling pathway, and pathway in cancer were all enriched in coexpressed genes in KEGG categories. Collectively, the increased expression of ADAMTS3 in the GBM could be linked to the vascular structure, ECM organization, and cancer‐related signaling.

In conclusion, we found that increased *ADAMTS3* expression in glioma stem cells is related to GBM aggressiveness and tumor development. We proposed that *ADAMTS3* is a potential target for glioblastoma therapy.

## 4 DISCUSSION

Glioblastoma Multiforme (GBM) is a type of brain tumor that is characterized by tumor infiltration into normal tissues, necrosis, cellular heterogeneity, and angiogenesis.^1^, ^2^ Cancer stem cells (CSCs), a type of cancer cell, are a major source of oncogenesis and render tumors resistant to chemo‐ and radiation.^3^, ^4^ As a result, we researched molecular components connected to cancer stem cells that were also expressed at a high level in the brain stem or progenitor cells.

A Disintegrin and Metalloproteinase with Thrombospondin Motifs (ADAMTS) enzymes are secreted matrix‐associated zinc metalloendopeptidases that play a variety of roles in tissue morphogenesis and pathological remodeling, inflammation, and vascular biology.^5^, ^6^ Cancer cells and stromal cells can both secrete these enzymes, and they may play a role in modifying the tumor microenvironment through a variety of methods.^7^, ^8^ Thus, ADAMTSs can cleave or interact with a wide range of extracellular matrix components or regulatory factors, regulating cell adhesion, migration, proliferation, and angiogenesis. Furthermore, Multiple ADAMTS genes have been found mutant, overexpressed, or epigenetically silenced in cancers of various origins, suggesting that these metalloproteases play a role in cancer genesis.^8^ Given the importance of metalloproteinases in the formation and progression of GBM,^20^, ^21^, ^22^ we examined the ADAMTS3 gene, which is increased in GSC and may have a role in tumor growth. ADAMTS3 has recently been shown to be able to activate VEGF(vascular endothelial growth factor)‐C in experimental animal models by proteolytic cleavage, via a method that is currently unknown but presumably involves CCBE1, a secreted protein with a collagenous domain.^18^, ^19^, ^23^ According to a prior study, the lymphatic vessel is widespread in glioma.^24^, ^25^ Especially, Meng FW et al discovered that nestin^+^ glioma stem cells differentiated into lymphatic vessels through histological analysis.^26^ However, how lymphatic vessel affects glioma malignancy remains unknown. VEGF‐C is the primary regulator of lymphangiogenesis in embryonic development and a variety of lymphangiogenic activities in adults.^23^ Furthermore, as recently discovered, VEGF‐C functions as an autocrine and paracrine pro‐survival cytokine, supporting neural stem cell, tumor cell survival, and tumorigenesis in many cancer cells.^27^, ^28^, ^29^, ^30^, ^31^ However, the relationship between ADAMTS3 and GBM has not been revealed. Therefore, in further study, we will find how the paracrine and autocrine signaling of GSC will affect lymphangiogenesis and tumor maintenance.

In this study, *ADAMTS3* expression is considerably downregulated during GSC differentiation in serum‐containing media. In orthotopic GBM mouse models, Lee et al. found that serum‐cultured cells lost their tumorigenic capacity.^16^ Accordingly, ADAMTS3 may promote malignancy by maintaining self‐renewal ability in GSCs. Consistent with this hypothesis, inhibiting ADAMTS3 expression using shRNA has decreased GSCs' tumorsphere formation capacity and cell proliferation. We also confirmed that the mRNA level of multiple glioma‐related stemness markers decreases when ADAMTS3 expression is suppressed. Furthermore, we have shown that ADAMTS3 knockdown cells lost their tumor formation ability in the orthotopic GBM mouse models. Hence, these findings indicate that ADAMTS3 expression is critical in maintaining tumorigenesis by ensuring self‐renewal and tumor initiation ability of GSCs.

Recent studies have revealed genes that can be used as prognostic markers in glioblastoma and glioma stem cells.^29^, ^30^, ^31^, ^32^ Likewise, ADAMTS3 is likely to be used as a prognostic marker like these studies. However, more research is needed to better understand the mechanisms of how ADAMTS3 maintains GSCs. It may be possible to improve patient diagnosis and prognosis predictions as well as the development of more efficient GBM treatments with further understanding of the function role and expression pattern of ADAMTS3 in GSCs.

## FUNDING INFORMATION

This research was supported by the National Research Foundation (NRF) funded by the Ministry of Science and ICT (MSIT) (no. 2021R1A2C1003561, 2022R1I1A3070961, and 2022R1A2C1011742).

## CONFLICT OF INTEREST

The authors declare no conflict of interest.

## Data Availability

The data that support the findings of this study are openly available in Gene Expression Omnibus; (GEO) at https://www.ncbi.nlm.nih.gov/geo/query/acc.cgi?acc=GSE4536, reference number.
